# Quantification and characterisation of commensal wild birds and their interactions with domestic ducks on a free-range farm in southwest France

**DOI:** 10.1038/s41598-022-13846-2

**Published:** 2022-06-13

**Authors:** Chloé Le Gall-Ladevèze, Claire Guinat, Pierre Fievet, Benjamin Vollot, Jean-Luc Guérin, Julien Cappelle, Guillaume Le Loc’h

**Affiliations:** 1 IHAP, ENVT, INRAE, Université de Toulouse, Toulouse, France; 2 Department of Biosystems Science and Engineering, ETHZürich, Mattenstrasse, Basel, Switzerland; 3 SIB, Lausanne, Switzerland; 4 ENVT, Toulouse, France; 5 Aigues-Vives, France; 6 ASTRE, CIRAD, INRAE, Université de Montpellier, Montpellier, France; 7 UMR ASTRE, CIRAD, 34398 Montpellier, France

**Keywords:** Ecological epidemiology, Ecological networks, Microbial ecology

## Abstract

The role of commensal birds in the epidemiology of pathogens in poultry farms remains unclear. Our study aimed to identify potential key species for interactions with domestic ducks on one free-range duck farm in southwest France. Methods combined direct individual observations on duck outdoor foraging areas, network analysis, and general linear mixed models of abundances. Results showed a wide diversity of wild bird species visiting foraging areas, heavily dominated in frequency by White wagtails (*Motacilla alba*) and Sparrows (*Passer domesticus* and *Passer montanus*). These also were the only species seen entering duck premises or perching on drinkers in the presence of ducks. Moreover, White wagtails were the species most frequently observed on the ground and in close proximity to ducks. Network analysis suggested the role of White wagtails and Sparrows in linking ducks to other wild birds on the farm. The abundance of White wagtails was positively associated with open vegetation, with the presence of ducks and particularly in the afternoon, while the abundance of Sparrows was positively associated only with the fall-winter season. By precisely characterising interactions, the study was able to identify few wild bird species which should be prioritized in infectious investigations at the interface with poultry.

## Introduction

Commensal wild birds are species that live permanently or temporarily near human activities, benefitting from food and shelter resources^1^. Such as the ubiquitous House Sparrow (*Passer domesticus*), they can be present in all types of human-made environments, ranging from town-centres to isolated farms^1–4^. Depending on the human activities considered, commensalism involves different profiles of species, according to their life history traits making them adapted to the specific resources from which they benefit and to the specific hazards that threaten them^3,5^. In poultry farms, and particularly free-range farms, the frequent presence of commensal wild birds alongside domestic poultry has raised concerns about possible transmission of pathogens such as avian influenza viruses (AIV) between wild and domestic bird communities^6,7^. While the main reservoir hosts for these viruses are waterbirds (Anseriformes and Charadriiformes)^8,9^, common commensal species such as Sparrows, Starlings and Pigeons have also been shown to be susceptible to AIV in experimental conditions^10–15^. Commensal birds could thus play a role in AIV transmission at the wild-domestic bird interface. Although previous surveillance studies have shown that commensal birds were less susceptible to AIV infection in comparison with waterbirds^8^, their mobile behaviour and frequent presence on poultry farms make them suitable candidates as bridge hosts, i.e. hosts providing a link through which AIV could be transmitted between poultry farms and with other wild bird populations^16^.

Southwest France is a poultry production region with a high density and diversity of species and breeding systems. Free-range systems such as traditional foie gras duck farms are particularly abundant in the region^17^. Free-range farming systems automatically increase the likelihood of contacts at the wild-domestic bird interface, especially for the slow growing foie gras ducks which live for at least 11 weeks with unlimited access to outdoor foraging areas. This exposure was recently called into question as the region, in particular its duck sector, was deeply affected by recurrent HPAI waves in the past few years^18–21^. Preventive contact-restriction measures have been consequently enforced on poultry farms to reduce the risk of direct and indirect AIV transmission at the wild-domestic bird interface. These measures are mainly based on covering drinkers and feeders, and food and litter storages, generally repelling the presence of wildlife on farms, and confining flocks during high-risk periods of AIV introduction^22^. However, these measures remain costly and sometimes inconsistent with traditional free-range production systems^23,24^. Their efficiency also has not yet been assessed as no quantitative data are available on wild bird visits and contacts with free-range poultry in southwest France^24^. Nevertheless, few publications have broadly described wild bird visiting patterns on poultry farms in the southwest and other regions in France^25–27^. The results show variations of wild bird communities between regions and seasons, with overall few to no visits of waterbirds and maximal abundances on farms for Sparrows (*Passer domesticus*), Finches (*Fringilla spp.*), Pigeons and Doves (*Columbidae*), Starlings (*Sturnus vulgaris*) and Magpies and Jackdaws (*Corvidae*). However, knowledge gaps remain with regard to an accurate quantification and characterisation of interspecies (wild-wild and wild-domestic) interactions involving commensal wild birds through their biological seasons. Moreover, it is very likely that declining rural avian biodiversity due to decades of agroecosystem changes^28,29^ has had an impact on interactions at the wild-domestic bird interface. Indeed, declining farmland specialist species may have left more space to thrive around poultry farms for the generalist commensal species that are better adapted to more homogenised agricultural landscapes^29,30^.

The present study aims to identify potential key commensal wild bird species in the avian host community that are in contact with domestic ducks. We do so by studying one typical duck farm in southwest France, combining direct individual observations, general linear mixed models of abundances, and network analysis. Specifically, the study focused on (1) describing wild bird species and their interactions with domestic ducks, (2) analysing the network of wild bird species co-occurrence with domestic ducks, and (3) investigating the environmental factors influencing wild bird species abundance.

## Methods

### Study site

**Figure 1:**
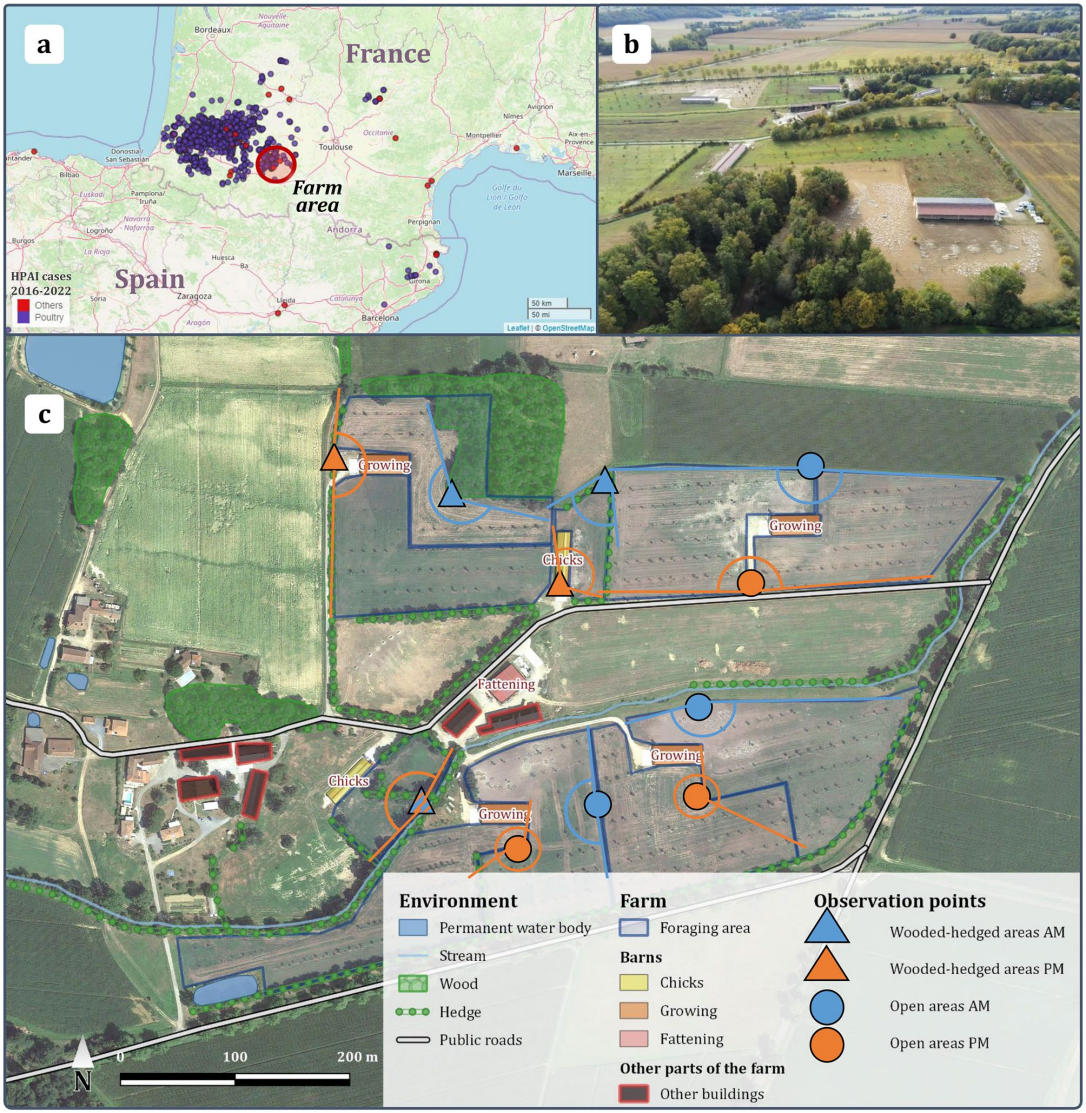
Environment of the study farm. (**a**) Geographic location of the farm (red circle) compared to locations of outbreaks and cases in poultry (purple) and wild birds (red) of highly pathogenic avian influenza (HPAI) from October 2016 to February 2022. Base map is the interactive HPAI map from Plateforme ESA accessible on https://shiny-public.anses.fr/shiny-vsi/ (April 2022). (**b**) North aerial view of the farm (drone photography taken by the authors). (**c**) Map of the farm showing the natural environment surrounding duck foraging areas. Unlabelled buildings are private houses that are not part of the farm. Observation points are marked on each studied area by type of surrounding environment (shape) and time of day (colour), with their field of view indicated as lines delimitating the angle connected by curved line (circle when a point is used for two areas). The map is produced using QGIS^31^ version 3.6.1-Noosa, with a base map from Google, Image © 2022 CNES/Airbus, Maxar Technologies.

The study site is a typical duck farm in the department of Gers, southwest France, which was affected by HPAI during the 2016–2017 winter epizooty (Fig. 1a). The surroundings of the farm are an undulating landscape of small mixed crop and stock farms with woods on hills (Fig. 1b). Although not a wetland area, many artificial water dams of various sizes are dispersed in the surroundings for agricultural use. The farm breeds mule ducks under the ‘Canard à Foie Gras du Sud-Ouest’ label, which requires at least 14 weeks of unlimited outdoor access. The farm is composed of two small 0.5 ha outdoor foraging areas for ducklings (1 day to 1 month of age), and eight large 1.5 ha foraging areas for growing ducks (1 month to 14–16 weeks of age) (Fig. 1c). Like an increasing number of poultry farms in the region, an agroforestry program is implemented on the farm, so trees for wood are planted on all outdoor foraging areas, and hedges of fruit trees are planted around some of the areas (Fig. 1b,c).

### Field observations of wild birds

From July 2020 to June 2021, standardised observations of wild birds were implemented on the farm’s outdoor foraging areas. An average of 8.7 (median of 8) observation sessions (each lasting one hour) were conducted each month 4 weeks apart (except in February and May due to the regulatory stamping out and cleaning for HPAI that took place on the farm) over two consecutive days. Generally, four sessions were distributed evenly over the day: half an hour after sunrise, mid-morning, mid-afternoon, and one hour before sunset. In total, 87 observation sessions were carried out during 20 days distributed over 10 months (see description in Supplementary Table S1). For each month, three of the 10 foraging areas were randomly selected to meet the following criteria: one with ducks present on it, one with no ducks for at least 2 weeks, and one with a recent change in use (installation or removal of ducks in the preceding 2 weeks). The three selected areas were then visited on an alternating basis over the 2 days of observation, so that each had at least one morning and one afternoon observation when possible, or at least two different times of day (see details in Supplementary Table S1). The same observation points were used for each foraging area, with only a regular adaptation to the direction of sunlight between morning and afternoon (Fig. 1c). Due to regulatory restrictions on duck movements between farms and on outdoor access during the high-risk period of HPAI introduction in the winter of 2020–2021, no area with a recent change in use was present in January 2021, and no area with presence of ducks in March, April and June 2021 (see Supplementary Table S1). Each observation session was conducted by the same person and consisted of 30 continuous left-to-right screenings of the area using binoculars to record individual observations of each wild bird present on a vertical projection of up to 10 m of the area including fences. For each bird, the following information was recorded (see details in Supplementary Table S2): species, behaviour and direct environment (location, type of perch or ground, proximity of less than 1 m from a domestic duck). If a bird could not be identified to the species level, all birds of the same genus or family were grouped together in the database. The observer also recorded the following environmental information: date, time, weather, presence and number of domestic ducks.

### Description of wild bird population diversity and behaviours

For each observation session, the maximum number of individuals observed per species during a single screening (i.e. at the same time) was recorded. This number was used as an estimation of the minimum number of individuals per species and per session. All sessions of the same month were then grouped together to calculate the monthly means of minimum number of individuals per species on the farm. The Shannon and Simpson diversity indices, and Piélou evenness index^32,33^ were calculated based on the monthly means rounded to the upper integer, using the “diversity” function of the “vegan” package on R software version 4.0.5^34^. The three indices were defined by the following formulae:

$$ {\text{H }} = {\text{exp}}\left( { - \mathop \sum \limits_{i = 1}^{S} p_{i} \ln p_{i} } \right)\quad \quad \left( {{\text{Shannon}}} \right); $$

$$ {\text{D}} = 1/\sum\limits_{1 = 1}^{S} {p_{i}^{2} \quad ({\text{Simpson}});} $$

$$    J = \frac{{\ln H}}{{\ln S}}\quad \left( \text{Pi}\acute{\text{e}}\text{lou} \right) $$

where $p_{i}$ is the proportion of individual birds in the species *i*, and *S* is the species richness (number of species), so that $\mathop \sum \limits_{i = 1}^{S} p_{i} = 1$. According to these definitions, the Shannon index represents the entropy in the population, i.e. the uncertainty in identifying the species of a random individual in the population. The Simpson index represents the diversity, i.e. the probability for two random individuals to belong to the same species. This second diversity index is less sensitive to rare species than the first one, so it is more suitable to compare populations from different contexts^32^. The Piélou index represents the equitability of species abundances, that is the evenness of distribution of individuals among the different species^32,33^.

The data subset of observations in presence of ducks on the area (for either more than 2 weeks or recently introduced, counting for 34 out of the 87 sessions) were used to quantify behaviours allowing contacts (direct or indirect) between wild birds and domestic ducks. Recordings of whether each wild bird was at a distance of less than one meter from a duck were used to assess direct contacts^35^, including droplet emissions (allowing airborne viral transmissions based on approximate droplet emission distance^36^ and virus infectivity in droplets^37^) or even physical contacts. Indirect contacts via biological matter (allowing indirect viral transmissions such as faeces, surfaces, water, or airborne particles) were assessed via recordings of bird locations on duck foraging areas, in particular around aggregation spots, such as duck premises, feeders, drinkers, and on trampled wet ground. The cumulative numbers of individuals showing each type of interaction with ducks (direct or indirect), or each type of behaviour (entering premises, on the ground or perched) were then compared between species or group of species.

### Network of between-species co-occurrences

To analyse the co-occurrence patterns (as a proxy for indirect contacts) between wild bird species and domestic ducks, data of observations on the 87 sessions with or without ducks on the area were used. An undirected weighted network was built, considering bird species (wild bird species or groups of species, and domestic ducks) as nodes, and two different bird species recorded during the same observation session (i.e., co-occurrence) as an edge. Edges were then weighted by the frequency at which a given pair of bird species was recorded. Each node was characterised by its degree, representing the number of other nodes (i.e., species) connected to it. Communities of more densely connected species^38^ were defined by a walktrap algorithm based on a random walk through the edges^39^ using the “cluster_walktrap” function of “igraph” package^40^, with a four-step walk selected as giving the most synthetic and biologically accurate result^39^. The network analysis was conducted using the “igraph” package^40^ on R software version 4.0.5^34^.

### Influence of environmental factors on wild species abundance

Environmental factors influencing the abundance of commensal wild birds on foraging areas were investigated, focusing on the species most at risk for interactions at the wild-domestic interface. To do so, counts of the most abundant species in presence of ducks were analysed according to environmental variables. Species selected for the analysis were the most frequently observed in presence of ducks, i.e. present on more than 25% of sessions with ducks in the area: White wagtails and Sparrows. This ensured also to exclude species with observation counts too low for analysis, i.e. too low for models to converge or to be interpretable. The following explanatory categorical variables were considered: time of day (morning or afternoon), weather (sun, clouds, or rain), season (spring–summer or fall-winter), type of surrounding vegetation of the area (woods and hedges, or open field) and presence of ducks on the area (either absent, or present in the last 2 weeks). All further statistical work was conducted using R software version 4.0.5^34^. As univariate distribution of abundances for both species showed no significant effect of weather (p-values over 0.7 for Kruskal–Wallis rank sum tests), this variable was excluded from model selections. Association between pairs of categorical explanatory variables was examined using chi-squared tests, considered significant if their p-value was greater than 0.05. The strength of this association was estimated by Pearson’s Phi using “phi” function from package “effectsize”^41^, and considered too large to keep both variables if their value was greater than 0.5^42^. Since species’ counts showed a negative binomial distribution, and to account for groups of sessions from the same 2-day visits of each month, general linear mixed-effects models using a log link negative binomial error distribution were used with the month as the random variable, using the “glmer.nb” function from “lme4” package^43,44^. Variable selection within the multivariate analysis was based on automated selection based on AIC using the function “dredge” from package “MuMIn”^45^, starting from a full model with all interactions between explanatory variables. Among minimal ranked models in a range of two units above the lowest AIC, the most parsimonious one was selected as the final model. The regression coefficients for the final model were expressed as odds ratios (OR) with 95% confidence intervals (CI), and its goodness of fit was estimated by the trigamma conditional R^2^^46^ using the “rsquared” function from package “piecewiseSEM”^47,48^.

## Results

### Description of wild bird population diversity and behaviours

A total of 42 wild bird species were observed on foraging areas of the farm during the 87 observation sessions and exactly 2000 individual observations (species-behaviour-environment); the vast majority belonged to the order *Passeriformes*. The most frequently observed species was White wagtail (*Motacilla alba*, MOTALB) in 84% of the total number of sessions, House and Tree Sparrows (*Passer domesticus* and *P. montanus*, PAS spp) in 79% of sessions, and Common Chaffinch (*Fringilla coelebs*, FRICOE) in 28% of sessions, while other species were observed on less than 25% of sessions. The minimal number of individuals (i.e. maximum observed at the same time) varied with species, and ranged up to 44 individuals for European Goldfinch (*Carduelis carduelis*, CARLIS). Resulting monthly means of minimum species group sizes ranged from 1 to 29.3, with a few increases in the number of gregarious species (Table 1).

**Table 1:**
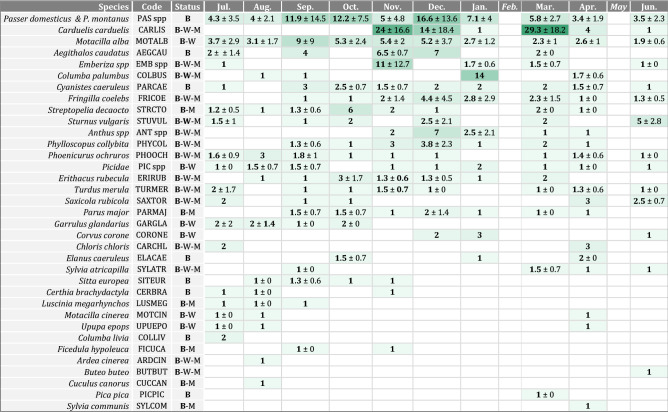
Monthly mean (± standard deviation) of minimum number of individuals per species, listed in descending order of cumulative means.

The darker the cell, the higher the mean. National population status of each species is taken from UICN France (2016)^49^, indicated as breeding (B), wintering (W), migrant (M). A combination of statuses is indicated if different populations are present along the year, and figured in bold if the population is consistently evaluated and significant for the species according to UICN’s criteria.

**Figure 2:**
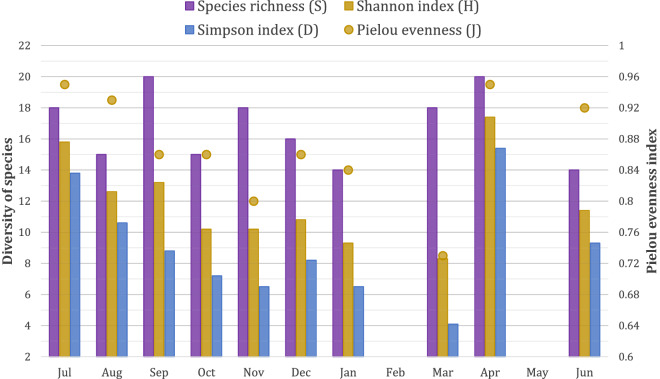
Monthly observed bird diversity indices on duck foraging areas.

The monthly number of wild bird species ranged from 14 to 20 (Fig. 2). The diversity indices of Simpson (D) and Shannon (H) and the evenness index of Piélou (J) (Fig. 2) showed similar patterns, with highest values in April (D = 15.4, H = 17.4, J = 0.95) and July (D = 13.8, H = 15.8, J = 0.95), then dropping during fall and winter down to the lowest values in March (D = 4.1, H = 8.3, J = 0.73).

**Figure 3:**
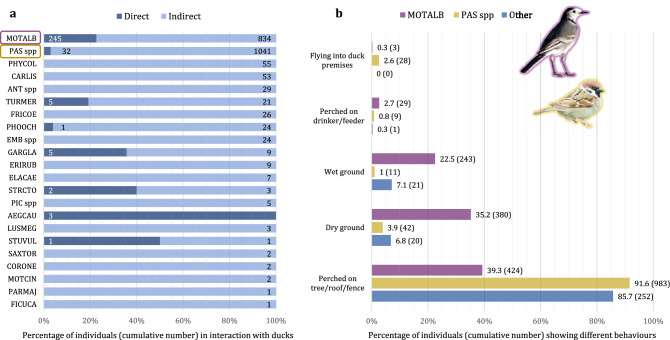
Distribution of cumulative numbers of individuals by species or species group involved in different types of interactions with domestic ducks over 953 observations of 2,446 cumulative individuals in total. (**a**) Distribution by proximity with ducks, considering a direct interaction as a distance to ducks closer than one meter (indirect otherwise). (**b**) Distribution by type of behaviour in the two most abundant species groups compared to all others. Species names are coded as follows: AEGCAU: *Aegithalos caudatus*; ANT spp: *Anthus spp*.; CARLIS: *Carduelis carduelis*; CORONE: *Corvus corone*; ELACAE: *Elanus caeruleus*; EMB spp: *Emberiza spp*.; ERIRUB: *Erithacus rubecula*; FICUCA: *Ficedula hypoleuca*; FRICOE: *Fringilla coelebs*; GARGLA: *Garrulus glandarius*; LUSMEG: *Luscinia megarhynchos*; MOTALB: *Motacilla alba*; MOTCIN: *Motacilla cinerea*; PARMAJ: *Parus major*; PAS spp.: *Passer domesticus* & *P. montanus*; PHOOCH: *Phoenicurus ochruros*; PHYCOL: *Phylloscopus collybita*; PIC spp.: *Picidae*; SAXTOR: *Saxicola rubicola*; STRCTO: *Streptopelia decaocto*; STUVUL: *Sturnus vulgaris*; TURMER: *Turdus merula*.

Using data of observations in presence of ducks, the direct environment of individual birds were analysed to quantify behaviours allowing potential direct or indirect interactions between wild birds and domestic ducks. Nine of the 23 species (8 of the 22 groups of species) observed in the presence of domestic ducks were recorded at least once at less than one meter from ducks (Fig. 3a). The frequency of these direct contacts represented 131 observations in the 953 total observations with presence of ducks (13.7%), implying a cumulative count of 294 individuals out of the 2446 cumulative individual birds. The vast majority of direct contacts implied MOTALB with 106 observations of 245 individuals, accounting for 83% of all birds showing direct contact with ducks and for 23% of all interactions with ducks shown by the species (Fig. 3a). This was followed by PAS spp, with 14 observations of 32 individuals, accounting for 11% of birds in direct contact with ducks and for 3% of interactions with ducks by the species (Fig. 3a). This was followed by *Turdus merula* TURMER and *Garrulus glandarius* GARGLA with each one 5 individuals (each one accounting for 1.7% of birds in direct contact, and respectively 19% and 36% of interactions by each species), followed by *Aegithalos caudatus* AEGCAU (3 individuals, 1.0% of birds in direct contact and 100% of interactions by the species), *Streptopelia decaocto* STRCTO (2 individuals, 0.7% of birds in direct contact and 40% of interactions by the species), and *Sturnus vulgaris* STUVUL and *Phoenicurus ochruros* PHOOCH (each one with 1 individual, 0.3% of birds in direct contact, and respectively 50% and 4% of interactions by each species) (Fig. 3a). Recordings of bird behaviour and locations on foraging areas revealed situations of proximity between wild birds and duck aggregation areas such as feeders, drinkers or inside duck premises (Fig. 3b). These observations (41 out of 953, implying a cumulative count of 70 individuals) implied only 3 species: MOTALB (25 observations of 32 individuals), PAS spp (15 observations of 37 individuals) and STRCTO (1 observation of one individual on a drinker). Only MOTALB and PAS spp were observed flying into duck premises, by 3 and 28 individuals (on 3 and 9 occasions), respectively (Fig. 3b). Contrary to other species, MOTALB were most of the time observed on the ground of duck foraging areas, on 272 observations (52% of 523 for the species) implying a cumulative number of 623 individuals out of 1079 (58%). Of these, 114 observations of 243 individuals (22%) were precisely on wet ground trampled by ducks (Fig. 3b).

### Network of between-species co-occurrences

**Figure 4:**
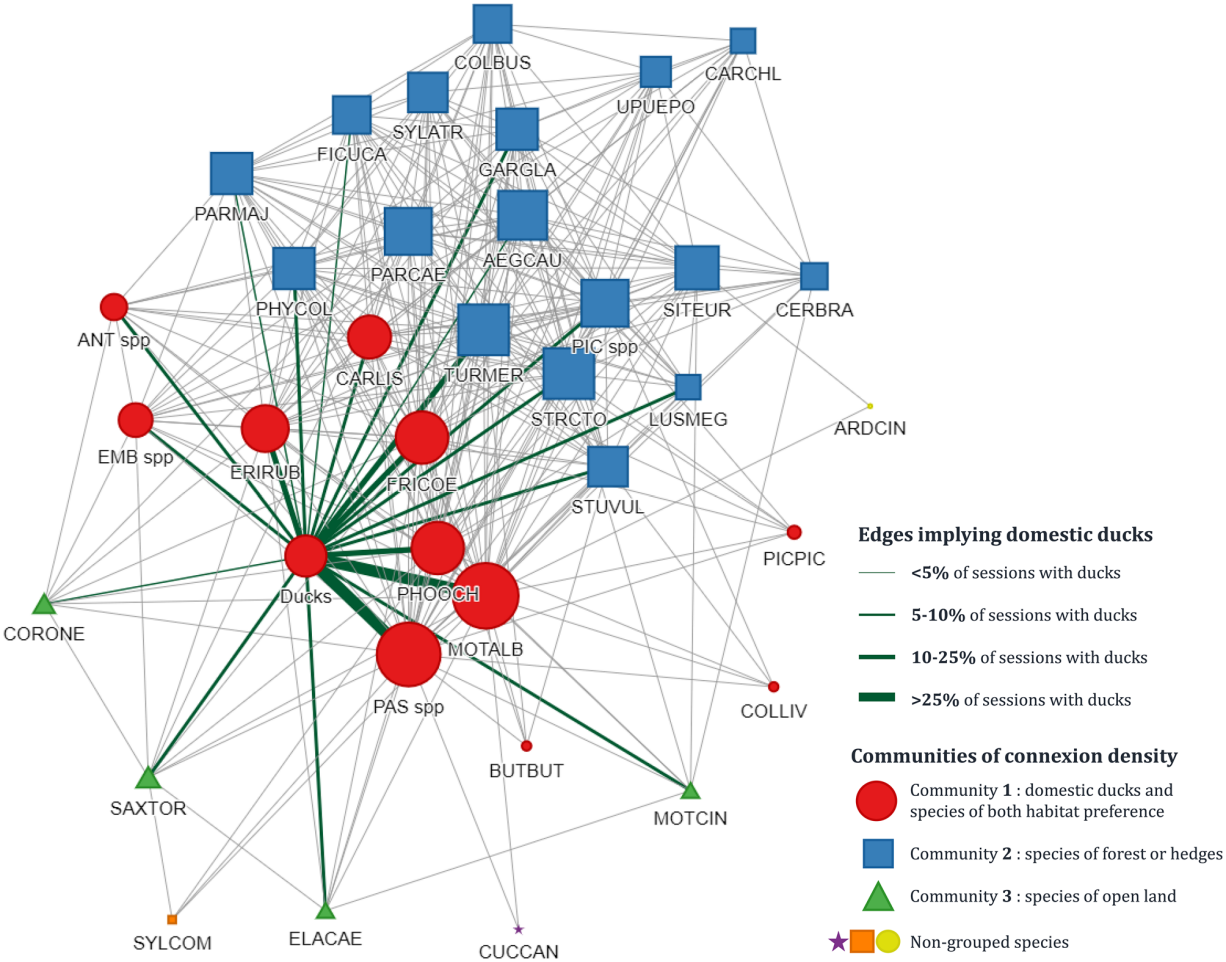
Undirected weighted network based on 87 observation sessions on duck foraging areas, leading to 1761 individual observations of 36 species or groups of species. Nodes are each species (or group), edges are observed interactions (co-occurrence) between two species. Nodes are coloured and shaped by community and sized by degree (number of contact-species), and arranged to show the most connexions as possible. Interactions with ducks are highlighted in green. Edge thickness is relative to their weight, i.e. their relative frequency in the total number of observation sessions. Species names are coded as follows: Ducks: Domestic ducks; AEGCAU: *Aegithalos caudatus*; ANT spp: *Anthus spp*.;ARDCIN: *Ardea cinerea*; BUTBUT: *Buteo buteo*; CARCHL: *Chloris chloris*; CARLIS: *Carduelis carduelis*; CERBRA: *Certhia brachydactyla*; COLBUS: *Columba palumbus*; COLLIV: *Columba livia*; CORONE: *Corvus corone*; CUCCAN: *Cuculus canorus*; ELACAE: *Elanus caeruleus*; EMB spp: *Emberiza spp*.; ERIRUB: *Erithacus rubecula*; FICUCA: *Ficedula hypoleuca*; FRICOE: *Fringilla coelebs*; GARGLA: *Garrulus glandarius*; LUSMEG: *Luscinia megarhynchos*; MOTALB: *Motacilla alba*; MOTCIN: *Motacilla cinerea*; PARCAE: *Cyanistes caeruleus*; PARMAJ: *Parus major*; PAS spp.: *Passer domesticus* & *P. montanus*; PHOOCH: *Phoenicurus ochruros*; PHYCOL: *Phylloscopus collybita*; PIC spp.: *Picidae*; PICPIC: *Pica pica*; SAXTOR: *Saxicola rubicola*; SITEUR: *Sitta europea*; STRCTO: *Streptopelia decaocto*; STUVUL: *Sturnus vulgaris*; SYLATR: *Sylvia atricapilla*; SYLCOM: *Sylvia communis*; TURMER: *Turdus merula*; UPUEPO: *Upupa epops*.

The undirected weighted network of between-species co-occurrences on the farm over 1 year was analysed. The network consisted of 36 nodes, one for domestic ducks and the other 35 for wild bird species (including 4 groups of species and 31 individual species) (Fig. 4). Nodes were connected by 323 edges representing the aggregation of 1217 observed co-occurrences of species pairs on the 87 selected observation sessions. Frequencies of co-occurrences (i.e., weight of edges) were very heterogeneous, with MOTALB and PAS spp paired on 74% of sessions as these species were observed on 85% and 80% of sessions, respectively. In contrast, other pairs of wild species were observed on less than 25% of all observation sessions. In presence of ducks on the area (34 observation sessions), only two species were co-occurring with ducks on more than 25% of sessions: MOTALB (94% of sessions) and PAS spp (85% of sessions). The two species were paired on 82% of sessions in presence of ducks. Other high frequencies of co-occurrences involved MOTALB with PHOOCH (23% of sessions), MOTALB or PAS spp with FRICOE (both 22% of sessions), and PAS spp with PHOOCH (21% of sessions). All other species’ pairs were observed on less than 19% of sessions. Degrees (i.e., number of paired species) were also heterogeneous, from MOTALB co-occurring with all other species and showing the highest degree of 35, to ARDCIN and CUCCAN that were rarely observed and both showing a degree of 2. PAS spp showed the second highest degree of 34, followed by FRICOE and PHOOCH with a lower degree of 28. As ducks were not present with all observed wild bird species, they showed a degree of 22. The walktrap algorithm identified three communities (numbered from one to three), including 12, 17, and 4 nodes, respectively (Fig. 4). Three species were not grouped with others by the algorithm. The three communities were representative of habitat preference of birds. Community 1 included domestic ducks and wild bird species of either wood or open land that were the most connected to ducks and their most-frequently visiting species. Communities 2 and 3 were composed of wild bird species living preferentially in woods or in open lands, respectively.

### Influence of environmental factors on wild species abundance

The abundances of MOTALB (present in 84% of observation sessions) and PAS spp (79%) were statistically analysed in relation with environmental factors. Presence of ducks was moderately and significantly associated with season, with more observation sessions without ducks in spring–summer season (X^2^ = 7.69, *p* = 5.55e−03, phi = 0.32). Presence of ducks was also significantly associated with surrounding vegetation, with a weaker association, due to more observation sessions without ducks in wooded-hedged areas (X^2^ = 4.49, *p* = 3.40e−02, phi = 0.25). Results of the general linear mixed-effects model (Table 2) showed that the number of MOTALB was positively and significantly associated with open areas in comparison with wooded-hedged areas (OR = 1.82, 95% confidence interval (CI): 1.33–2.48, *p* = 1.60e−04). The effect of time of day alone was weak and not significant, but it was stronger and significant in interaction with duck presence, with more abundance of MOTALB on the afternoon with ducks on the area in comparison with the morning or without ducks (OR = 2.00, 95% CI: 1.06–3.77, *p* = 3.26e−02). Abundance of MOTALB was also positively and significantly associated with the presence of ducks on the area (OR = 2.01, 95% CI: 1.27–3.17, *p* = 2.71e−03). The fitted model explained 52.3% of data variance according to the trigamma conditional R^2^. As for PAS spp, the general linear mixed-effects model showed that counts were only positively and significantly associated with fall-winter in comparison with spring–summer (OR = 2.60, 95% CI: 1.64–4.17, *p* = 5.64e−05) (Table 2). The fitted model was much less predictive with 9.8% of explained variance according to the trigamma conditional R^2^.

**Table 2:** Results of the negative binomial general linear mixed-effects models for White wagtails and Sparrows on duck foraging areas.

Species	Environmental variables		Cumulative counts	OR	Confidence interval [2.5–97.5%]	*p*-value	Conditionnal R^2^	AIC of fitted model (AIC of full model)
White wagtails (MOTALB)	Vegetation	Wooded Open	103 191	*Reference* **1.82**	1.33–2.48	1.60e−04	0.523	347.4 (360.0)
	Time of day	Morning Afternoon	105 189	*Reference* 0.97	0.57–1.63	8.98e−01		
	Duck presence	Absent Present	68 226	*Reference* **2.01**	1.27–3.17	2.71e−03		
	Duck presence :Time of day	Absent or morning Present on the afternoon	132 162	*Reference* **2.00**	1.06–3.77	3.26e−02		
Sparrows (PAS spp)	Season	Spring–Summer Fall-Winter	165 309	*Reference* **2.60**	1.64–4.17	5.64e−05	0.098	472.0 (488.7)

For each species, counts per session are the response variable, and effects of explanatory environmental variables according to the minimal fitted model are detailed. For both species, the full model from which the AIC is given used the formula *Logit(counts)* = Vegetation*Season*Time of day*Duck presence with the Month as random effect.

Significant values are in [bold].

## Discussion

Detailed counts and individual observations of wild birds visiting outdoor foraging areas of a typical free-range duck farm made it possible to characterise the species diversity of the avian commensal population visiting duck facilities, as well as to quantify different types of direct and indirect interactions with duck flocks. This study of wild bird interactions with free-range poultry is to our knowledge the most detailed one after few previous studies^50–54^, with the combination of year-long series of data, species-level identification and individual behaviour quantification. The originality of this study lies in the multi-species contact network analysis applied to the context of a wildlife-livestock interface^55^, in addition with the focus on a free-range duck farm that represents a specific but least-studied type of poultry production.

The farm studied is not directly connected to any wetland or littoral areas, which makes it theoretically unattractive for wild aquatic birds such as Anseriformes, Charadriiformes or Ardeidae that are considered as main reservoirs for AIV^8,9^. Indeed, results showed that the vast majority of visiting wild birds were non-aquatic species (except for one Grey heron, *Ardea cinerea*). Most of them were passerines, which allowed to specifically focus this study on terrestrial non-aquatic avian communities of such farm environments. The observed species richness and diversity on foraging areas is slightly lower than periurban areas of France 20 years ago^56^, or comparable to most types of agricultural lands in France^29^. However, the actual diversity of the farm is certainly greater than what was observed in this study, as farm areas outside duck foraging areas were specifically excluded, and only visual counts were applied. Indeed, the surrounding vegetation (woods, riparian vegetation, hedges) and habitats (various crops, duck premises, barns, old houses and gardens) of the whole farm are attractive for a vast diversity of wild birds throughout the year, as suggested by a study in the Netherlands^50^ and as seen on visits for other purposes and on nest captures on the study farm (20–30 species, H = 36.6–54.6, unpublished data). Interestingly, the profile of bird species observed on our study farm was quite different from other studies. Columbidae, Corvidae and Sturnidae were observed on less than 21% of observation sessions, and mostly in small numbers, while they composed a large part of observations in studies on other world regions and were thus identified as important species^7,27,51,53,54^. This difference in visiting bird species highlights the importance of studying wild-domestic bird interface in each environmental context of interest. Thus, infectious risk analyses can be focused on species that are the most abundant and in close interaction with domestic birds, such as White wagtails and Sparrows in the context of this study.

Co-occurrence and behaviour analyses together revealed the key role of White wagtails and House and Tree sparrows in the multi-species interaction network surrounding free-range domestic ducks. These three species were by far the most frequent and abundant species either when ducks were present or not, all year long, and with a resulting high number of species in indirect contact (co-occurring in the same area). It appears that White wagtails and Sparrows can play a role as a link (at least by indirect contacts on duck foraging areas) between ducks and other wild birds that may less frequently visit ducks, thus intensifying the global connection between the wild and domestic compartments of the local interface. The three species, and to a lesser extent other species of the duck-centred community (number 1), seem to intensify local connections between communities of birds that are usually observed in distinct habitats such as woods or open lands. Moreover, White wagtails were observed in co-occurrence with the only aquatic bird (Grey heron) observed during this study on an area with no ducks on it. As White wagtails are very mobile and omnipresent on the farm, it is highly probable that on the same day (or few surrounding days if considering AIV incubation similar to Sparrows^10–13^) the same individuals went in direct or indirect contact with other species of wild or domestic birds of the farm. White wagtails may then help connect domestic ducks and the community of commensal birds of the farm to more distant and less frequent species that may be more susceptible to infections. Such epidemiological connection between White wagtails and European starlings, another commensal bird species of poultry farms, has already been suggested by a previous study in the same region^57^. In addition to their abundance, White wagtails and Sparrows showed bold behaviours towards ducks and their facilities, frequently approaching ducks at a close distance, perching on their drinkers and feeders, flying into their premises, and foraging on trampled grounds rich in duck faeces. The one-meter distance chosen here was an estimation of potential direct airborne transmission via droplets applied to various respiratory infectious agents, including AIV^35^. It was based on approximate droplet emission distance^36^ and virus infectivity in droplets^37^. Although observations of wild birds in close proximity to ducks were overall infrequent, they constituted the only evidence of possible direct infectious transmission between wild birds and ducks, as physical contacts were never observed in this study, and have rarely been observed on poultry farms in other studies^51,54^. In addition to direct contact occasions, the presence of White wagtails and Sparrows on drinkers, feeders, inside duck premises or on wet trampled ground made indirect transmission of potential infectious agents from and to ducks highly possible. This risk has to be further evaluated for these particular species of wild birds. While Sparrows are commonly observed in most other studies of poultry farms^7,25,27,54^, and are known for their susceptibility to AIV infection^10–13^, they are sedentary and probably purely commensal hosts that may only transmit infectious agents at a very local scale. White wagtails are rarely considered in epidemiological studies around poultry farms^6,54,58,59^; however, the species is much more mobile than Sparrows, both on a daily basis^60^ and seasonally, with partial migratory behaviour and winter aggregations in the region of this study^61^. Besides farms, the species is detected in a large diversity of habitats, and it is particularly attracted by wetlands, foraging on open areas along river or lake banks where wild aquatic birds can be present (data from French Breeding Bird Survey^62^). White wagtails are thus excellent candidates as bridge hosts^16^ for pathogens shared between domestic ducks and water birds, such as AIV, as suggested in a previous multi-site study^7^. This role on the study farm, which serves as a model of farms in southwest France, needs to be further evaluated through local ecological and epidemiological studies.

Analysis of environmental factors favouring abundance of Sparrows showed a higher concentration of birds during the fall and winter seasons. Either by population increase (young birds of the year from the local breeding population), or by aggregation on foraging areas rather than other environments, this element certainly increases their role in intensifying local connections between communities of birds. This intensification of connections is of particular concern for future epidemiological studies as it happens at a time of year when persistence of infectious agents in the environment is favoured by low temperatures and high humidity^63–65^, and when highly pathogenic AIV are regularly at risk of being introduced in poultry farms of the region by migratory water birds^19,66^. At the scale of the study farm, Sparrows seemed to have no preference in terms of weather, time of day or surrounding vegetation. This reveals their constant presence under all conditions around duck areas, probably due to their granivore feeding behaviour associated with their sedentary behaviour around farm buildings where they build colony shelters. In contrast, White wagtails were slightly more abundant in the afternoon and consistently more abundant when ducks were present or on areas that were not surrounded by hedges or woods. The species therefore seems to require more specific types of environments than Sparrows, and probably also chooses the best time of day to forage on duck areas when arthropods are more active. Indeed, most of the time they were observed on the ground hunting for arthropods that are numerous around duck manure and litter^67^. Again, further ecological studies have to be carried out in the region to find out what other sites are visited by White wagtails when they are not observed on the farm. However, duck presence and open-vegetation areas showed a weak positive association that might increase the effect of one variable or the other in the model. Moreover, duck presence showed a moderate positive association with fall-winter season, which also needs to be taken into account in conclusions. Therefore, abundance of White wagtail may be positively influenced by fall-winter season in parallel with duck presence, and abundance of Sparrows may be positively influenced by duck presence in parallel with fall-winter season.

Practices of the studied farm are very likely representative of most regions of southwest France, thus allowing to extrapolate a good part of the conclusions, provided that local bird communities are similar. In particular, the farm has been applying daily preventive measures that are recommended by sanitary authorities for several years: covering feeders and drinkers, repelling wildlife on the farm with electric fences and rodent poisoning, checking for and removing dead domestic ducks every day. However, it should be noted that from January 2021 the presence of ducks in the outdoor areas was disturbed as the farm was under regulatory restrictions due to regional control measures for the HPAI H5N8 epizooty that occurred in the winter of 2020–2021. Consequently, no recent introduction or removal of ducks happened on the farm in January, then preventive culling in February (no observation) led to the absence of ducks during the months which followed in the spring of 2021, allowing vegetation to grow on all foraging areas. These changes in surface use and activity on the farm may have impacted the presence of commensal birds. This idea seems to be supported by the lowest diversity and evenness indices in March, as abundances were dominated by few species (mostly groups of CARLIS, and few MOTALB and PAS spp) that comply with the cleaning activities that were occurring on most parts of the farm. With growing vegetation and less activity during spring, these changes possibly reverted in favour of less farm-dependent species along with the ascending migratory visitors, making the diversity indices rise in April.

As the aim of this study was to describe commensal birds, the observation protocol had to focus on foraging areas only. If observation biases hid some elusive or hardly identifiable species, these could be considered as occasional or distant visitors rather than truly commensal. It is certain that the most important species targeted in terms of numbers and duck-centred behaviours were certainly not missed by the protocol implemented in this study, although the sanitary disturbances described above might have lowered their numbers in the spring of 2021. However, in order to be more exhaustive regarding bird species that visit the farm, a study at night time could be useful as domestic ducks have access to foraging areas during the night and nocturnal birds (owls and snipes for instance) were detected on some occasions during visits for other purposes. To detect a broader range of species and complete the assessment of targets for future studies, the observation method implemented in this study could then be complemented by other methods like nocturnal cameras^51,54^ or identification of bird DNA accumulated in environmental samples on domestic duck areas^68^. These methods could provide large benefits and should be considered in further research.

In a context of declining farmland bird populations in France and Europe^28,69^, it is interesting to highlight the presence of particularly threatened species in our study site. According to evaluation of French breeding bird populations^49,69^, two of the species observed on foraging areas of the farm are declining steeply and are classified as endangered on the national IUCN Red List: *Emberiza schoeniclus* (− 50% 2001–2019 decline), and *Passer montanus* (− 60%). Five other species also are declining steeply but continue to be classified as vulnerable on this list: *Anthus pratensis* (− 66%), *Chloris chloris* (− 50%), *Carduelis carduelis* (− 31%), *Dryobates minor* (− 31%), and *Emberiza citrinella* (− 54%). It therefore appears important to preserve in particular the consequent nesting population of *Passer montanus* thriving on this farm thanks to shelters in old buildings and rich sources of food brought by diverse agricultural activities and natural habitats.

The overwhelming presence of White wagtails and Sparrows observed throughout the year on the farm, and their frequent and close proximity with domestic ducks, raises questions concerning the efficacy of contact-restricting measures. Indeed, the ability of these commensal species to adapt to complex environments driven by human activities makes them virtually impossible to strictly keep away from free-range poultry farms. In addition, in traditional free-range systems such as the one studied here, sustainable measures have to preserve the freedom of domestic ducks as much as possible, but also preserve endangered farmland birds by having as little unwanted impact as possible. Some wildlife-preventing systems with variable efficacy and costs are being tested on poultry farms and other agricultural facilities^59,70^: nets, laser^71^, balloons, kites^72^, propane canons, and scaring alarm calls^73^. While they may keep the most fearful and larger species away (particularly aquatic birds), the boldest ones might still eventually find a way to access the attractive places on farms where animals live, either by habituation^71^ or by taking advantage of inherent flaws or defects in the system. To avoid habituation, costly strict wildlife-prevention measures should be used for short periods of time and when absolutely needed. Moreover, most contact-restriction measures are aimed at preventing poultry from being infected by wildlife, even though it has been proven to be a minor introduction risk in previous HPAI epizooties^17,74^. Contact-restriction measures often do not consider the environmental transmission risk from poultry to wildlife, but waste water, feathers, dust and aerosols coming from thousands of infected birds on a farm can represent a massive contamination source for commensal wild birds living in the same environment^75–77^. After characterising what and how wild-domestic interactions occur and identifying a few species that could represent ecological bridges, it is necessary to assess whether these bridges are epidemiologically functional as well^78^. In a second phase, considered commensal species have to be characterised with regard to their infectious susceptibility and excretion capacity for the pathogen of interest, such as HPAI. Effectively infected commensal wild birds could then spread the infectious agent to other farms, or to a wild maintenance host. Without complete knowledge of the bridge host potential of every commensal species for every infectious agent, and as a precautionary measure, overall biosecurity measures on poultry farms stay useful and need to consist of multiple complementary measures. They should prevent wild birds from coming into contact with poultry or their facilities, but also poultry-contaminated materials (litter, manure, waste water, dust) from being accessible to wild birds and vice versa (natural watercourse or water body, water run-off, litter and feeding materials). It is of first importance to take into account all possible dispersion pathways to limit the risk of spillover and spillback events between wild and domestic birds, in particular for highly transmissible, rapidly evolving and devastating agents such as HPAI.

## Supplementary Information

Supplementary Information.

## Data Availability

The datasets generated and analysed during the current study are available from the corresponding author on reasonable request.
