# Supplementary material for: Quantification and characterisation of commensal wild birds and their interactions with domestic ducks on a free-range farm in southwest France
Source: Sci Rep. 2022 Jun 13;12:9764. doi: 10.1038/s41598-022-13846-2 (PMC9192735; doi:10.1038/s41598-022-13846-2)
Supplement: Supplementary file 1 — Supplementary Information. [file 41598_2022_13846_MOESM1_ESM.docx]

Quantification and characterisation of commensal wild birds and their interactions with domestic ducks on a free-range farm in southwest France

Chloé Le Gall-Ladevèze ^1^, Claire Guinat ^2,3^, Pierre Fievet ^4^, Benjamin Vollot ^5^, Jean Luc Guérin ^1^, Julien Cappelle ^6,7^, Guillaume Le Loc’h ^1,^*

1. IHAP, ENVT, INRAE, Université de Toulouse, Toulouse, France
2. D-BSSE, ETH Zürich, Switzerland
3. SIB, Lausanne, Switzerland
4. ENVT, Toulouse, France
5. Independent researcher, Aigues-Vives, France
6. ASTRE, CIRAD, INRAE, Université de Montpellier, Montpellier, France
7. CIRAD, UMR ASTRE, F-34398 Montpellier, France

* Corresponding author (email: guillaume.leloch@envt.fr)

**Table S1** - Distribution of the 87 observation sessions by month, time of day and presence of duck flock in the area. From January 2021, the presence of ducks in the outdoor areas was disturbed as the farm was under regulatory restrictions due to regional control measures for the highly pathogenic avian influenza H5N8 epizooty. **^a^** First months of observation consisted of more sessions to optimise the logistic of recording data and distribution of observation times. **^b^** Due to logistical needs in October and November 2020, the two sessions in absence of ducks were on early and late morning with no session in the afternoon for this type of area. **^c^** Total number of sessions in presence or recent change of duck flocks, that were grouped as a unique level of explanatory variable in the analysis of wild bird abundances.

|  |  | **Duck flock in the area** | | | **Number of sessions by month and time** | |
| --- | --- | --- | --- | --- | --- | --- |
| **Month** | **Time** | **Presence** | **Recent change** | **Absence** |  |  |
| July 2020 | AM | 3 | 1 | 3 | **7** | **^a^** |
|  | PM | 4 | 3 | 1 | **8** | **^a^** |
| August 2020 | AM | 1 | 2 | 3 | **6** | **^a^** |
|  | PM | 3 | 1 | 2 | **6** | **^a^** |
| September 2020 | AM | 1 | 1 | 2 | **4** | |
|  | PM | 1 | 2 | 1 | **4** | |
| October 2020 | AM | 1 | 1 | 2 ^b^ | **4** | |
|  | PM | 2 | 1 | - | **3** | |
| November 2020 | AM | 1 | 1 | 2 ^b^ | **4** | |
|  | PM | 1 | 1 | - | **2** | |
| December 2020 | AM | 1 | 2 | 1 | **4** | |
|  | PM | 1 | 2 | 1 | **4** | |
| January 2021 | AM | 2 | - | 2 | **4** | |
|  | PM | 2 | - | 1 | **3** | |
| March 2021 | AM | - | - | 4 | **4** | |
|  | PM | - | - | 4 | **4** | |
| April 2021 | AM | - | - | 4 | **4** | |
|  | PM | - | - | 4 | **4** | |
| June 2021 | AM | - | - | 4 | **4** | |
|  | PM | - | - | 4 | **4** | |
| **Number of sessions by duck flock presence in the area** | | **24** | **18** | **45** | **87** | |
|  |  | **42 ^c^** | |  |  | |

**Table S2** – Description of recorded information during observation sessions

|  | Variable | Levels |
| --- | --- | --- |
| General data for the observation session | **Observation point** | Indication of one of the 11 points, as showed on Figure 1 |
|  | **Date** | Date of observation session |
|  | **Time** | Starting time of observation session |
|  | **Presence of ducks** | Absent for more than two weeks, present for more than two weeks, absent for less than two weeks, present for less than two weeks |
|  | **Weather** | Sun, clouds, rain |
| Individual bird data | **Screening** | Rank of screening over the area when the bird is detected |
|  | **Species** | Identification of species or species group of the bird |
|  | **Number** | Number of birds of the same species, on the same environment, and with the same behaviour that are present during the same screening |
|  | **Behaviour** | Perched on tree, perched on roof, perched on fence, perched on feeder, perched on drinker, on wet ground, on dry ground, flying into premises |
|  | **Proximity to ducks** | Whether or not at least one duck is present at less than one meter |
|  | **Comments and details** | Possible comments to report any problem, any change of environment during the session (like human presence) or to provide details that might help further data analysis |
